# Combined Thickness and Permittivity Measurement of Thin Layers with Open-Ended Coaxial Probes †

**DOI:** 10.3390/s19081765

**Published:** 2019-04-12

**Authors:** Kjetil Folgerø, Kjetil Haukalid, Jan Kocbach, Andreas Soto Peterson

**Affiliations:** 1 NORCE Norwegian Research Centre AS, Fantoftvegen 38, NO-5892 Bergen, Norway; kjha@norceresearch.no (K.H.); jako@norceresearch.no (J.K.); 2 Department of Physics and Technology, University of Bergen, Allégaten 55, NO-5007 Bergen, Norway; Andreas.Peterson@uib.no

**Keywords:** open-ended coaxial probe, permittivity, thickness measurement, finite element method

## Abstract

This paper presents a method to simultaneously determine the thickness and permittivity of thin layers from multi-frequency reflection coefficient measurements using an open-ended coaxial probe. This is achieved by exploiting that the probe becomes radiating at frequencies higher than the probe’s typical operating range. Permittivity information is extracted from measurements in the typical frequency range, whereas thickness information is obtained from high frequency measurements by exploiting resonances that occur when the radiated waves are reflected at the layer boundary. A finite element model of the measurement set-up is made in COMSOL Multiphysics^TM^, and a matrix of simulations spanning the relevant layer thicknesses and permittivity range is generated. The measured permittivity spectra of unknown samples are compared to the simulation matrix to estimate layer thickness and permittivity. The method is verified by measurements of water–ethanol mixtures. An application example where the water fraction and layer thickness of a gas hydrate deposition layer is estimated from permittivity measurements in a multiphase flow loop is also presented.

## 1. Introduction

Dielectric spectroscopy is a method well suited to characterize mixtures of water and other substances. The open-ended coaxial probe [1,2,3,4,5,6,7,8,9,10] is suited for non-intrusive permittivity measurements of fluids flowing in pipelines as the sensor can be placed flush with the inner pipe wall and thereby not disturb the flow. In many applications, the material to be characterized has a limited thickness [9,10]. Examples are early detection and monitoring of growth of scale depositions on a pipe wall and fouling in heat exchangers, as well as water fraction measurements of films of condensed water in wet gas transportation systems. In these cases, the probe will sense an effective permittivity that depends on the thickness of the dielectric layer and the permittivity of both the dielectric layer and the backing material. The objective of this paper, which is an extension of a conference paper [11], is to present a methodology for obtaining both layer permittivity and layer thickness of relatively thin dielectric layers from measurements with open-ended coaxial probes. The focus is on liquid layers and deposits that grow directly on the probe, such that good contact between the probe and the material under test is achieved. Thus, any air gaps between the probe and the material under test leading to large errors in measured permittivity [10] are avoided.

The methodology described is of particular interest for detecting and characterizing unwanted deposition layers that can occur during pipeline transportation of multiphase mixtures of oil, gas, and water. In such systems, ice-like structures called gas hydrates may form at low temperatures and high pressures (see e.g., [12]). These gas hydrates may form deposition layers on the pipe wall, and in a worst case these layers can grow and eventually completely block the pipe. This is a huge flow assurance issue in the petroleum industry and great care is therefore taken to avoid gas hydrate deposition and plugging [13]. As of today, there do not exist reliable methods for detecting and characterizing such hydrate deposits. In previous work, we have shown that information about the gas hydrate properties, e.g., water content, can be obtained from the permittivity if the thickness of the layer is sufficiently thick [14,15]. In this paper, it will be shown how also the thickness of hydrate layers can be estimated from the measured permittivity spectrum.

The permittivity is a complex variable typically defined relative to the permittivity of vacuum

(1) $$\varepsilon_{0}\varepsilon^{*}=\varepsilon_{0}\left(\varepsilon^{\prime }-i\varepsilon^{\prime \prime }\right),$$

where $\varepsilon_{0}$ is the permittivity of vacuum, $\varepsilon^{*}$ is the complex relative permittivity with real ($\varepsilon^{\prime }$) and imaginary ($\varepsilon^{\prime \prime }$) parts, and i is the imaginary unit. In this work the term permittivity is used to refer to the term complex relative permittivity. The real and imaginary parts of the permittivity are referred to as dielectric constant ($\varepsilon^{\prime }$) and dielectric loss factor ($\varepsilon^{\prime \prime }$) respectively.

There exist full-wave analytical models for the coaxial probe terminated into layered materials reported in literature (e.g., [9,10]). These models can be used to establish a relation between the probe impedance and the layer permittivity and thickness. From the measured impedance (or reflection coefficient), the layer permittivity and thickness are found by solving the inverse problem. The approach chosen in this work is somewhat different, as the layered structure is considered as an effective medium. The effective permittivity of the structure is determined from measurements using a model assuming an infinite thickness. Thereafter, the layer permittivity and thickness are found from the effective permittivity using finite element method (FEM)modelling. The advantage of using FEM modelling instead of analytical full-wave models is that it is easily adapted to new and rather complex measurement situations.

**Figure 1:**
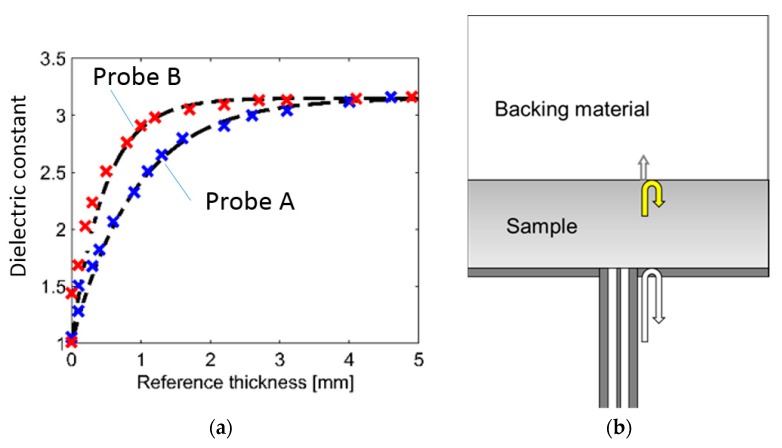
(**a**) Measured effective dielectric constant of an ice layer backed by air as a function of layer thickness. The diameter of Probe B is smaller than that of probe A. (**b**) Illustration of measurement problem: The permittivity of a sample of finite thickness backed by a backing material is measured using an on open-ended coaxial probe.

Permittivity measurements with the open-ended coaxial probe rely on analyzing the reflection from the probe-sample boundary. For samples thinner than (approximately) the probe outer conductor radius, the measured effective permittivity will be a function of the permittivities of the sample and the backing material, the sample thickness, and the probe dimensions [16]. The effective permittivity can to a first order approximation be described by an empirical relation,

(2) $$\varepsilon_{empiric}^{*}=\left(\varepsilon_{2}^{*}-\varepsilon_{1}^{*}\right)e^{\left(-\frac{d}{D}\right)}+\varepsilon_{1}^{*},$$

where $\varepsilon_{1}^{*}$ is the permittivity of the sample, $\varepsilon_{2}^{*}$ is the permittivity of the backing material, d is the sample thickness, and D is an empirical probe constant dependent on the probe geometry. Figure 1a shows the measured effective permittivity of ice layers backed by air together with the calculated empirical effective permittivity. Results are shown for two probes with different geometry, i.e., different probe constants *D*.

**Figure 2:**
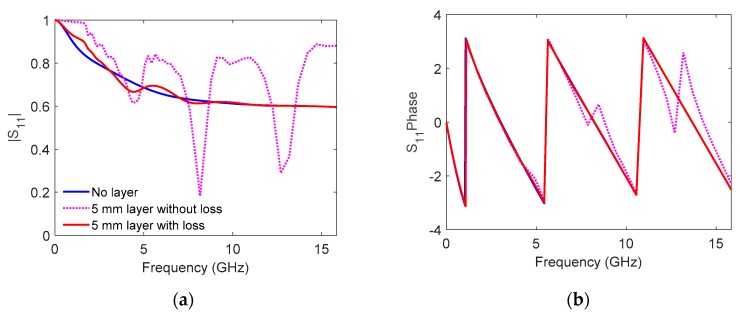
(**a**) Magnitude and (**b**) phase of simulated reflection coefficient for three water layers. Blue: Infinite thick layer. Red: 5 mm thick layer. Magenta: 5 mm thick layer, with water dielectric loss factor set to zero in the simulations.

The open-ended probe is usually applied for frequencies where radiation from the probe is negligible. However, the open-ended coaxial probe is known to become radiating at high frequencies (when the probe dimensions are comparable to the wavelength in the material under test). When measuring samples with finite thickness, this may result in additional reflections from the sample-backing boundary interfering with the main reflection from the probe–sample boundary (see Figure 1b). In Figure 2, COMSOL Multiphysics^TM^ (COMSOL AB, Stockholm, Sweden) [17] has been used to simulate the reflection coefficient for three different water layers: One infinite thick layer and two 5 mm thick layers, where one of the layers is modelled without dielectric loss. For the two thin layers, resonances appear in the simulated reflection coefficient due to the reflections from sample-backing boundary. This can be observed both in the magnitude and the phase of the reflection coefficient. In real measurements, the dielectric loss will dampen the reflections significantly, causing spectrum similar to the red spectrum in Figure 2. Nevertheless, if the permittivity calculation model applied assumes an infinite sample thickness, the additional reflections may result in artifacts in the effective (measured) permittivity. Thus, the empirical model in Equation (2) does not apply. Typically, the artifacts will be stronger at some frequencies due to resonance effects. The strength of these resonances and the resonance frequency depend on the sample thickness, the permittivity of the sample, the permittivity of backing material, and the dimensions of the probe. While such resonances most often represent an unwanted measurement error, they also contain information about the layer thickness and the layer permittivity. Hence, open-ended coaxial probes can be used for combined thickness and permittivity measurements.

## 2. Materials and Methods

### 2.1. Methodology

The method presented in this paper is based on comparison of the measured permittivity spectra with a matrix of FEM simulations that spans the relevant layer thickness and layer permittivity ranges. The FEM simulation software COMSOL Multiphysics [17] has been used to build a two-dimensional axisymmetric model of the open-ended coaxial probe and dielectric layers used in the experiments. The resonances occur in the GHz region for the studied probe.

After all simulations are completed, the matrix is transformed to the same frequency axis as the measured spectra using interpolation techniques. The layer thickness and permittivity can then be estimated by direct comparison of a measured spectrum and the simulation matrix by finding the layer thickness and layer permittivity whose simulated spectrum gives the best match to the measured spectrum. Note that instead of comparing measured and simulated permittivity values, the comparison can be done on simulated and measured reflection coefficients. The main advantage of working with permittivity is that the artifacts are clearly identified visually.

The method necessitates a priori knowledge of the backing material permittivity and the frequency variation (dispersion frequencies) of the layer permittivity. Unique solutions are obtained by exploiting that the relaxation of the dielectric layer is known through mixture models. For many applications, the layer is a mixture of water and one or more materials with very low permittivity and very weak dielectric dispersions, as compared to water. For such applications, the layer permittivity can be simulated and spanned using a two-phase mixing formula with water and the other material(s) permittivities as input. If large temperature variations are expected, the simulation matrix should also span the expected temperature range, since the permittivity of water shows large variations with temperature in the frequency range where the resonances occur.

In order to test and verify the method, measurements and simulations of mixtures of ethanol and water were performed. The permittivity of ethanol–water mixtures is well characterized by Petong et al. [18]. For the gas hydrate application example discussed later, Hanai’s model [19] is used to calculate the effective permittivity of gas hydrates with free liquid water.

### 2.2. Experimental

**Figure 3:**
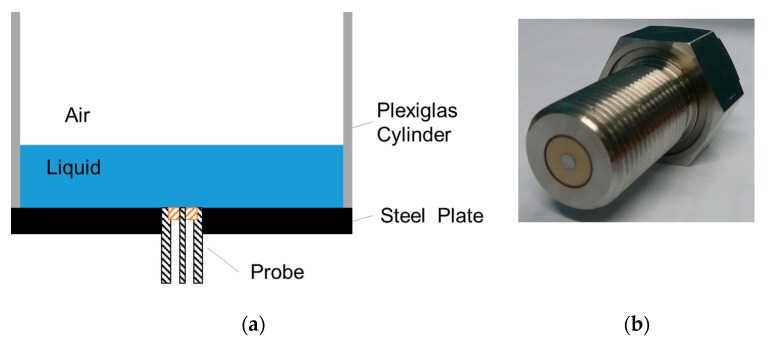
(**a**) Sketch of the experimental set-up and (**b**) picture of the open-ended coaxial probe used in the experiments.

Figure 3 shows a sketch of the experimental set-up and a picture of the open-ended coaxial probe used in this work. Various ethanol–water mixtures were poured into a liquid container, which was constructed by gluing a plexiglass cylinder to a steel plate. The probe was mounted through a threaded hole in the steel plate such that the end of the probe was flush with the bottom plane of the liquid container. The thickness of the liquid layers was controlled by adding a known volume of liquid to the container.

The open-ended probe has an inner and outer conductor radius of 1.5 mm and 5 mm, respectively. The probe is made of stainless steel with a plastic material between the inner and outer conductor. The probe was connected to a Rohde & Schwarz ZVL13 (Rohde & Schwarz, Munich, Germany) vector network analyzer (VNA) through a high-quality coaxial cable. The reflection coefficient was measured at 201 frequencies from 10 MHz to 14 GHz, with a higher density of frequency points above 1 GHz. The radio frequency power and intermediate frequency bandwidth of the VNA were −10 dBm and 1 kHz, respectively. The permittivity was calculated from measured reflection coefficients using the bilinear calibration procedure as described in [16].

### 2.3. FEM Simulations

A 2-dimensional axisymmetric model of the experimental set-up was made in COMSOL Multiphysics and solved using the RF module [17]. The probe’s internal structure and the liquid container with liquid layer was modelled. The permittivity of a range of mole fractions of ethanol in water was calculated by interpolating the dielectric relaxation parameters reported in [18]. Simulations of the reflection coefficient were then performed for these layer permittivities backed by air (i.e., permittivity of 1.0) for a range of layer thicknesses from 0 to 7 mm in steps of 0.1 mm. The permittivity was calculated from the simulated reflection coefficients using the bilinear calibration procedure as described in [16].

## 3. Results and Discussion

### 3.1. Sensitivity Considerations

**Figure 4:**
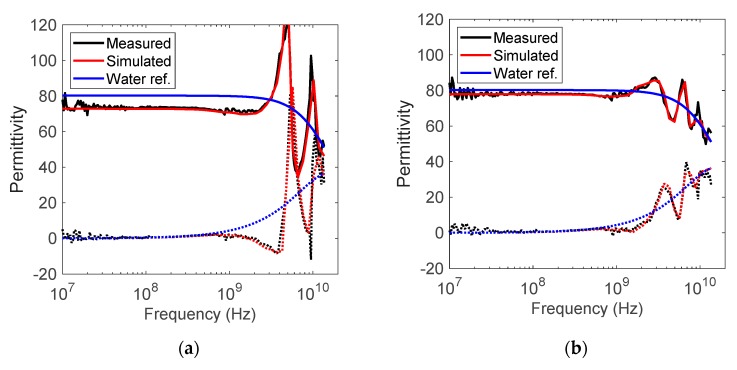
Comparison of measured and simulated relative permittivity spectra for liquid water layers. Liquid water reference spectrum is included. The dielectric constant is plotted with solid lines and the dielectric loss factor with dotted lines. (**a**) 3 mm water layer backed by air (**b**) 5 mm water layer backed by air.

Figure 4 compares measured and simulated permittivity spectra for thin water layers (3 and 4 mm thickness) backed by air. The simulated spectra in general fit well with the measured spectra, verifying the COMSOL Multiphysics simulations and the accuracy of the permittivity measurements. The artifacts in permittivity for the layers are clearly observed. The difference in response between the various layer thicknesses can also clearly be observed. The strength of the resonances decreases, and the first resonance moves towards lower frequencies with increasing layer thickness. As the artifacts in permittivity are not equally strong for all layer thicknesses and layer permittivities, the operating range for a probe with given geometry will be limited.

**Figure 5:**
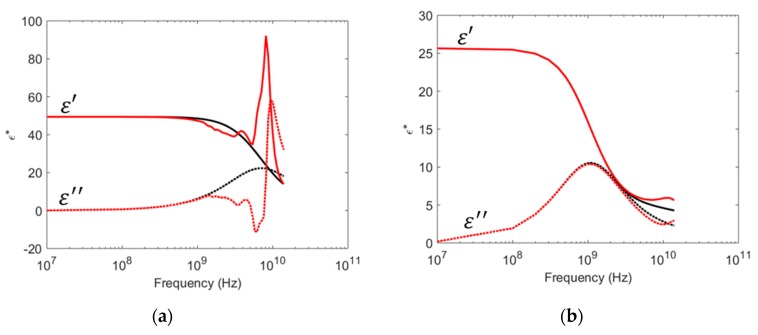
Comparison of the simulated relative permittivity spectra (red) and the empirical model given in Equation (2) of relative permittivity (black) for two liquid layers. The dielectric constant is plotted with solid lines and the dielectric loss factor with dotted lines. (**a**) 1.5 mm thick layer of 0.1 mol ethanol in water backed by air (**b**) 3 mm thick layer of 0.865 mol ethanol in water backed by air.

Figure 5a compares the simulated permittivity spectra for a 1.5 mm thick ethanol–water mixture layer (molar fraction of ethanol is 10%) with the permittivity estimated using the empirical model given in Equation (2). Similarly, Figure 5b compares the simulated and empirical model permittivity spectra for a 3 mm thick ethanol–water mixture layer (molar fraction of ethanol is 86.5%). This illustrates that the empirical model describes the permittivity response well at low frequencies, but fails at high frequencies when radiation from the probe causes layer reflections that interact with the reflections from the probe–sample interface. This difference is exploited to calculate the layer thickness.

The artifacts observed for the 3 mm ethanol–water layer in Figure 5b are much smaller than seen for the 3 mm water layer in Figure 4a. The reason for this is a combination of three factors: (1) The probe radiation increases with increasing sample dielectric constant, hence less energy is radiated into the ethanol–water layer than into the water layer. (2) The dielectric contrast between the layer and the backing material is weaker for the ethanol layer, causing a weaker reflection from the layer/backing boundary. (3) The dielectric loss factor is larger for water–ethanol mixtures than for pure water mixtures for frequencies below approximately 2 GHz, giving a larger attenuation of the radiated and reflected wave at these frequencies. All in all, this results in less artifacts in the mixture measurements than in the pure water measurements. As the attenuation of the radiated signal is small through a thin dielectric layer, the artifacts will be larger for thinner layers. This is the case for the 1.5 mm ethanol–water layer shown in Figure 5a.

As illustrated Figure 4 and Figure 5, the sensitivity of the methodology varies with layer thickness and layer permittivity. A measure of the sensitivity is found by calculating the difference between simulated effective permittivity and the effective permittivity estimated using Equation (2)

(3) $$\left|\Delta \varepsilon_{eff}^{*}\right|=\sum_{freq}\left(\left|\varepsilon_{sim}^{\prime }-\varepsilon_{empiric}^{\prime }\right|+\left|\varepsilon_{sim}^{\prime \prime }-\varepsilon_{empiric}^{\prime \prime }\right|\right),$$

with the permittivity of water–ethanol mixtures as layer permittivity and the permittivity of air as backing material.

**Figure 6:**
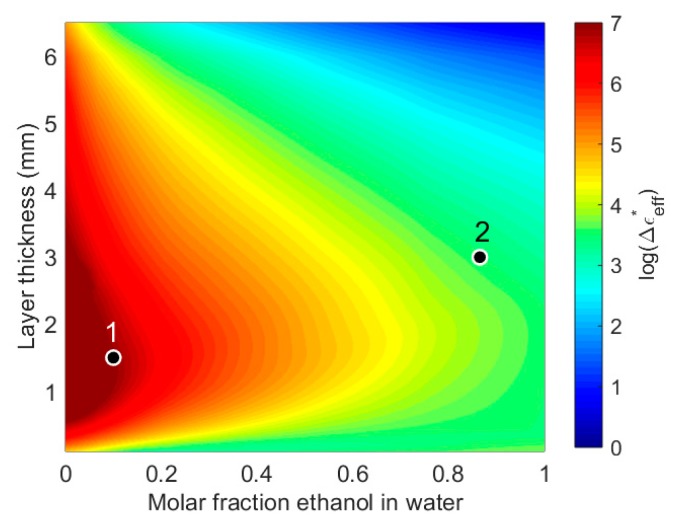
Plot of permittivity difference between simulated effective permittivity and empirical model as calculated by Equation (3). The frequency responses for the liquid layers marked 1 and 2 are shown in Figure 5a,b, respectively.

The sensitivity mapping is shown in Figure 6. The highest sensitivity for the studied probe is for liquid layers with high water content and thicknesses from 0.5 to 3 mm. The sensitivity then drops towards zero for thick layers of pure ethanol. The examples from Figure 5a,b are marked as 1 and 2 in the sensitivity plot, respectively. It is clearly observed that point 1 has a much higher sensitivity than point 2.

As discussed in the introduction, the strength of the resonances and artifacts will also depend on the dimensions of the probe. The simulations and measurements presented in this work are for a probe with inner and outer conductor radius of 1.5 mm and 5 mm, respectively. This probe radiates in the GHz range, whereas a smaller probe would radiate at higher frequencies. The sensitivity range for a given probe can be found by using sensitivity mapping such as shown in Figure 6.

To summarize, the method is best suited for combined thickness and permittivity measurement in cases where the resonances are large. This occurs for dielectric layers with the following properties: (i) high dielectric constant (as this results in high radiation from the probe), (ii) low dielectric loss factor and rather small thickness (such that the attenuation of the reflected signal is low), and (iii) high contrast in dielectric properties between the dielectric layer and the backing material (such that the reflected signal is strong).

### 3.2. Verification of Methodology

The inverse problem is to find the permittivity and layer thickness of an unknown sample from the measured permittivity. A 4 mm thick water layer and a 3 mm thick ethanol–water with mole fraction ethanol 86.5% are used as examples. The mixture layer corresponds to the permittivity spectrum in Figure 5b. A matrix of simulations spanning the mole fraction from 0 to 100% ethanol and the thickness range from 0 to 7 mm were obtained using COMSOL Multiphysics. The measured permittivity spectrum of the “unknown” sample is then compared to all simulated frequency responses, and the discrepancy between the measured and simulated response for each thickness and mole-fraction combination is calculated as

(4) $$\left|\Delta \varepsilon_{eff,ij}^{*}\right|=\sum_{freq}\left(\left|\varepsilon_{meas}^{\prime }-\varepsilon_{sim,ij}^{\prime }\left|+\right|\varepsilon_{meas}^{\prime \prime }-\varepsilon_{sim,ij}^{\prime \prime }\right|\right),$$

where *i* is the mole fraction index and *j* is the thickness index.

**Figure 7:**
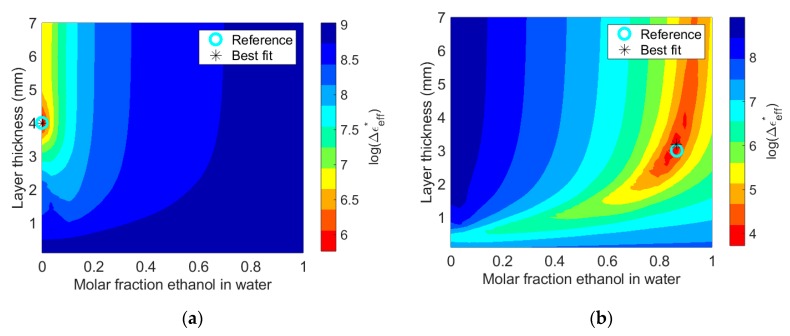
Plot of permittivity difference between measured permittivity and simulated permittivity as calculated by Equation (4). (**a**) 4 mm water layer (χ=0). (**b**) 3 mm ethanol–water layer with χ=0.865.

Figure 7 shows contour plots of the discrepancy between measured and simulated spectra for the 4 mm water layer and the 3 mm ethanol–water layer. The contour plots span layer thickness and molar fraction of ethanol in water. The best fit is close to the actual layer thickness and permittivity for both layers. For the water layer, the red colored area around the best fit (with low deviation from the simulated spectra) is much smaller than for the ethanol–water layer. This is due to stronger artifacts in the 4 mm water spectrum as compared to the 3 mm ethanol–water spectrum, giving lower uncertainty in the estimated layer thickness and permittivity for the water layer. This example illustrates that the uncertainty of the method varies with permittivity and layer thickness.

### 3.3. Application Example

**Figure 8:**
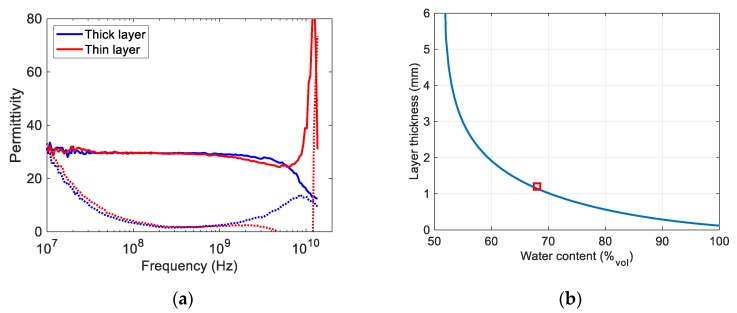
(**a**) Measured permittivity of two hydrate layers (**b**) Thickness and water content combinations that corresponds to the low frequency permittivity part of the spectrum. The best fit from the COMSOL Multiphysics simulations is shown as a red square.

**Figure 9:**
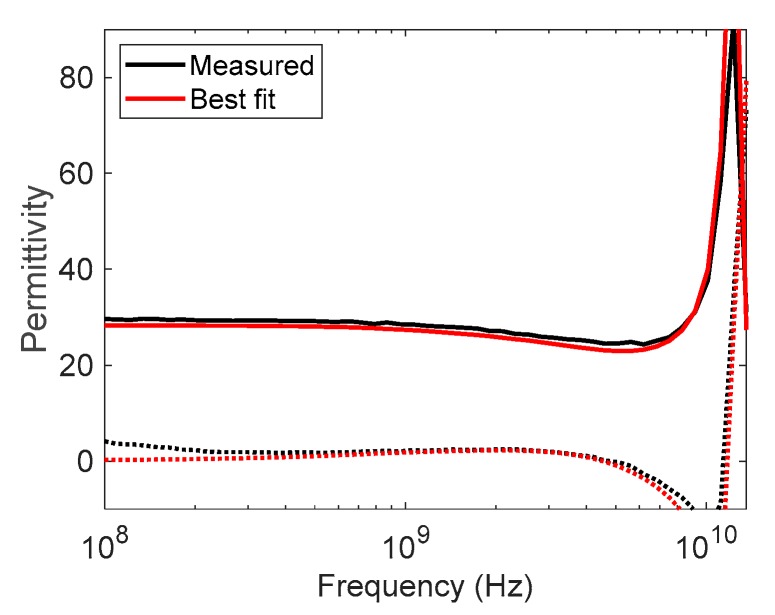
Measured permittivity of a hydrate layer backed by gas and best fit from COMSOL Multiphysics simulations.

A permittivity sensor was installed in a multiphase gas hydrate flow test loop with the probe end flush with the inner wall of the pipe. The permittivity was measured while water and gas formed a gas hydrate layer at the wall of the pipe. This layer will typically include a large amount of free water [20]. As discussed in previous work [1,21,22], the water content in the hydrate layer can be estimated using Hanai’s model [19] if the layer permittivity is known. Figure 8a shows the measured permittivity spectra for two hydrate layers with different thickness and water content. The low frequency responses of the two layers are similar, and it is not possible to extract information about both layer thickness and water content from this part of the spectra. Figure 8b shows the different combinations of thickness and water content that corresponds to the low frequency permittivity spectrum. This figure was calculated using the empirical model (2) in combination with Hanai’s model assuming a water permittivity of 80 and a hydrate permittivity of 3.5. As there are no artifacts in the “thick layer” permittivity, it can be deduced from FEM simulations that the thickness is larger than approximately 5–6 mm. From Figure 8b, it is found that this corresponds to a water content slightly below 55% by volume. To estimate the thickness of the layer for the “thin layer”, a simulation matrix was made using COMSOL Multiphysics spanning mixtures of water and gas hydrates according to Hanai’s model and thicknesses from 0 to 7 mm. Figure 9 shows the best fit of these simulations to the measured permittivity. This fit corresponds to a layer thickness of 1.2 mm with water content of approximately 68% by volume. A marker (red square) showing this point is included in Figure 8b.

## 4. Conclusions

In this paper, it has been shown that both sample permittivity and sample thickness can be estimated by comparing simulated and measured permittivity spectra obtained with an open-ended coaxial probe. The method was verified by measurement and simulations of liquid layers with known properties and thicknesses. An application example showed that the methodology is applicable for estimating water content and thickness of gas hydrate deposition layers in multiphase flow. The main limitation of the method is that the accuracy varies with layer permittivity and thickness, and a given probe will therefore have a restricted operating range.

## 5. Patents

The combined permittivity and layer thickness measurement method is protected by patents GB2527794B, US10,139,215B2 and patent application EP15814128.3A.
